# The impact of prenatal exposure to parasitic infections and to anthelminthic treatment on antibody responses to routine immunisations given in infancy: Secondary analysis of a randomised controlled trial

**DOI:** 10.1371/journal.pntd.0005213

**Published:** 2017-02-08

**Authors:** Stephen Nash, Alexander J. Mentzer, Swaib A. Lule, Dennison Kizito, Gaby Smits, Fiona R. M. van der Klis, Alison M. Elliott

**Affiliations:** 1 London School of Hygiene & Tropical Medicine, London, United Kingdom; 2 Wellcome Trust Centre for Human Genetics, University of Oxford, Oxford, United Kingdom; 3 The Jenner Institute, University of Oxford, Oxford, United Kingdom; 4 MRC/UVRI Uganda Research Unit on AIDS, Entebbe, Uganda; 5 Netherlands National Institute for Public Health and the Environment, Bilthoven, The Netherlands; George Washington University School of Medicine and Health Sciences, UNITED STATES

## Abstract

**Background:**

Chronic parasitic infections are associated with active immunomodulation which may include by-stander effects on unrelated antigens. It has been suggested that pre-natal exposure to parasitic infections in the mother impacts immunological development in the fetus and hence the offspring’s response to vaccines, and that control of parasitic infection among pregnant women will therefore be beneficial.

**Methodology/Principal findings:**

We used new data from the Entebbe Mother and Baby Study, a trial of anthelminthic treatment during pregnancy conducted in Uganda, to further investigate this hypothesis. 2705 mothers were investigated for parasitic infections and then randomised to albendazole (400mg) versus placebo and praziquantel (40mg/kg) during pregnancy in a factorial design. All mothers received sulfadoxine/pyrimethamine for presumptive treatment of malaria. Offspring received Expanded Programme on Immunisation vaccines at birth, six, 10 and 14 weeks. New data on antibody levels to diphtheria toxin, three pertussis antigens, Haemophilus influenzae type B (HiB) and Hepatitis B, measured at one year (April 2004 –May 2007) from 1379 infants were analysed for this report. Additional observational analyses relating maternal infections to infant vaccine responses were also conducted. Helminth infections were highly prevalent amongst mothers (hookworm 43.1%, *Mansonella* 20.9%, *Schistosoma mansoni* 17.3%, *Strongyloides* 11.7%, *Trichuris* 8.1%) and 9.4% had malaria at enrolment. In the trial analysis we found no overall effect of either anthelminthic intervention on the measured infant vaccine responses. In observational analyses, no species was associated with suppressed responses. Strongyloidiasis was associated with enhanced responses to pertussis toxin, HiB and Hep B vaccine antigens.

**Conclusions/Significance:**

Our results do not support the hypothesis that routine anthelminthic treatment during pregnancy has a benefit for the infant’s vaccine response, or that maternal helminth infection has a net suppressive effect on the offspring’s response to vaccines.

**Trial Registration:**

ISRCTN.com ISRCTN32849447

## Author summary

Parasitic infections, such as worms and malaria, have potent effects on the human immune system. These effects include modification of immune responses in the fetus and infant if a mother has a parasitic infection during pregnancy. These immunological changes can influence the way a child responds to the same infection when exposed in later life. It has been suggested that the immunological changes might also influence how the child responds to the vaccines given in infancy, and that treating mothers for parasitic infections when they are pregnant might be helpful. In this study we compared responses to vaccines between infants of mothers who had, or had not, been treated for worms while they were pregnant. We found no overall differences. We also compared vaccine responses between groups of mothers with and without parasitic infections. We found no evidence that the parasitic infections were associated with reduced responses in the children. This means that, although treating worms during pregnancy may have some benefits, improvements in the children’s responses to vaccines are not likely to be among them.

## Introduction

There is substantial evidence that pre-natal exposures are important in shaping immunological development [1]. This includes strong evidence that prenatal exposure and sensitisation to parasite antigens determines susceptibility to the same parasite in the offspring [1] and that immunisation during pregnancy influences the infant response to the same vaccine [2]. There is also evidence that prenatal exposures may influence the offspring’s response to unrelated antigens [1]. It is important to better understand such effects since they are likely to be important in broadly determining susceptibility to infectious diseases, either directly or through responses to immunisation, as well as determining susceptibility to other immunologically mediated conditions (such as allergy-related disease [3, 4]). Vaccines provide an example of a standardised immunological challenge given at a standardised time and hence an opportunity to evaluate the effects of pre-natal exposures on infant immune responses.

Recently, Malhotra and colleagues reported a study among children of mothers infected or uninfected with malaria and helminths in a coastal region of Kenya which suggested that infants of parasite-infected mothers had a reduced ability to develop antibody responses to *Haemophilus influenzae* type B (HiB) immunisation and diphtheria toxoid (DT), but showed no effect on responses to hepatitis B (Hep B) immunisation or tetanus toxoid (TT) [5].

If there is a causal association between prenatal parasitic exposure and infant vaccine responses, then treatment of maternal parasitic infections might be expected to remove parasite-associated effects. We conducted a randomised controlled trial (the Entebbe Mother and Baby Study, ISRCTN32849447) to investigate whether anthelmintic treatment of pregnant mothers improved the vaccine response amongst their children [6]. We have previously reported the effects of treatment on cellular responses following BCG and tetanus immunisation, and on tetanus and measles antibody concentrations: there were no overall effects but, in planned subgroup analyses, albendazole treatment of mothers with hookworm was associated with reduced T-helper 2 cytokine responses to TT in their infants, and (unexpectedly) albendazole treatment of mothers without hookworm resulted in increased interferon-γ(IFN-γ) responses to mycobacterial antigen; otherwise no effects of maternal treatment on responses to BCG, TT or measles were observed [7]. Here, we report on the effects of maternal anthelminthic treatment on a further six serological responses (DT, pertussis [pertussis toxin (PT), filamentous haemagglutinin (FHA) and pertactin], Hep B and HiB).

In addition to the trial results, we also present an observational analysis of associations between multiple maternal infections and infant immunological responses, analogous to the observational analyses reported by Malhotra and colleagues, using our existing dataset to investigate whether similar associations are also present in our cohort of mothers and babies from rural and urban areas of central Uganda.

## Methods

### Study design and participants

Healthy pregnant mothers in their second or third trimester were enrolled as part of the Entebbe Mother and Baby Study (EMaBS; ISRCTN32849447) between 2003 and 2005, described elsewhere [6, 7]. Briefly, this was a randomised, placebo-controlled, factorial study of the effect of single-dose albendazole (400 mg) and praziquantel (40 mg/kg) given during the second or third trimester of pregnancy on postnatal outcomes. Mothers were enrolled at their first antenatal visit unless they attended in the first trimester, in which case enrolment was postponed to minimise risk of teratogenicity. After enrolment they continued to receive standard antenatal care, including intermittent presumptive treatment for malaria with sulfadoxine/pyrimethamine and tetanus immunisation, and intrapartum and neonatal single-dose nevirapine for prevention of mother-to-child HIV transmission for the minority in whom it was required. Infants received the routine EPI (Expanded Programme on Immunisation) vaccines (BCG and polio at birth; DT, pertussis toxin, TT, Hep B and HiB at age six, 10 and 14 weeks, measles at nine months). All mothers gave informed written consent on behalf on themselves and their children. We have previously reported on responses to vaccines against tuberculosis, TT and measles [7]. Here we assess six immunological responses amongst children at age one year: DT, Hep B, pertussis, FHA, pertactin, Hep B, and HiB. Only children who received all three doses of pentavalent vaccine are included in this analysis.

Ethical consent was granted for the original trial and for subsequent analysis from UVRI (GC/127/12/07/32), the Uganda National Council for Science and Technology (MV625), London School of Hygiene & Tropical Medicine (790, A340), and the Oxford Tropical Research Ethics Committee (39–12).

### Maternal and infant infection status

At screening during pregnancy, and at delivery, or as soon as possible after delivery (for those whose children were born outside hospital), blood samples were obtained from each woman to test for presence of malaria parasites by thick film and for microfilariae of *Mansonella perstans* using a modified Knott’s method [8]; a single stool sample was obtained for diagnosis of intestinal parasites including hookworm (*Necator americanus* in this area [9]), *Schistosoma mansoni*, *Trichuris trichiura* and *Ascaris lumbricoides* using the Kato Katz technique [10] and for *Strongyloides stercoralis* by culture [11].

In case of multiple births just the first child was considered for inclusion in this analysis. Mother-baby pairs were excluded if the infant did not receive the standard three doses of EPI vaccines before samples were collected.

At one-year of age a blood sample was obtained from infants. Plasma and serum were separated and stored at -80°C until processing. Plasma or serum were assessed for antibody concentrations against DT, pertussis antigens and HiB using a Luminex bead-based multiplex immunoassay described in detail elsewhere[12, 13]. Antibody concentrations against Hep B were measured using the ABBOTT Architect i2000 with their anti-HBs kit (Abbott Laboratories, Chicago IL, USA) using the recommended protocol. We elected to measure the unstimulated serological response to vaccination in order to maintain consistency with other published reports of helminth-vaccine response associations. In the cases of DT and HiB these measures are likely correlated with protection against disease. In the case of pertussis it remains unclear which antigen is responsible for inducing protection and whether serological levels are sufficient correlates, whereas for hepatitis B, there is evidence that measuring peak response of antibody (approximately 6 weeks post final vaccination) is the optimal correlate of protection although practically this is very difficult to achieve [14].

Mothers were categorised by parasite exposure in three ways, following the approach of Malhotra 2015 for comparability [5]. First, mothers were grouped according to the total number of infections (helminths and malaria: 0; 1; 2; ≥3). Second, mothers were grouped as no infection; malaria only; malaria plus one helminth infection; malaria plus two or more helminth infections. Third, mothers without malaria were grouped as follows: no helminth infections; one infection and no malaria, two infections and no malaria; three or more infections and no malaria.

### Statistical analysis

The vaccines examined here were not the primary outcomes for this trial, so sample size calculations were not based on these responses. In a post-hoc evaluation of power, based upon the standard deviations we observed, we had 80% power to detect differences ranging from 1.18 (FHA) to 1.34 (HiB). A complete case analysis was done where possible, and imputation of missing data was not performed. Vaccine responses from the included and excluded records were compared with t-tests or Mann-Whitney tests, as appropriate.

As this was a factorial trial, comparisons were made between all those randomised to albendazole versus those randomised to matching placebo, and between praziquantel versus placebo. Linear regression was used to assess associations between exposures (albendazole and praziquantel) and outcomes (infant vaccine responses). Outcome variables were transformed onto the log (base 10) scale to reduce skew; hence reported coefficients represent geometric mean ratios (GMR). Regression was performed with a bias-corrected bootstrap using 100 replicates. Covariates were selected *a priori* and included maternal baseline characteristics of age, parity (1; 2–4; ≥5), education level (none; primary; secondary; tertiary), and household socio-economic group (on a six point scale, with six representing the highest group); infant covariates were sex, infant malaria and time (in days) since the third EPI vaccination. Pre-planned sub-group analysis was carried out to examine the effect of albendazole on children of mothers who had a hookworm infection, and separately for the effect of praziquantel on the children of mothers with *S*. *mansoni* infection. This was performed using the same regression technique as described above and allowing an interaction between randomised treatment and infection. Interaction effects between the two randomised treatments were tested in a similar way.

A similar approach was used to assess individual infections (including binary indicator variables for malaria, hookworm, *S*. *mansoni*, *M*. *perstans*, *Ascaris*, *Trichuris* and strongyloidiasis in one linear regression model) and the exposure categories defined above. In each case we also adjusted for randomised treatment. Exposure groups were treated as categorical variables designed to allow for a comparison with the previously published results from mothers in Kenya [5].

No adjustment was made for the numerous testing caused by assessing the effect of multiple exposures on six outcomes. Stata version 14.1 was used for all analyses.

## Results

**Fig 1:**
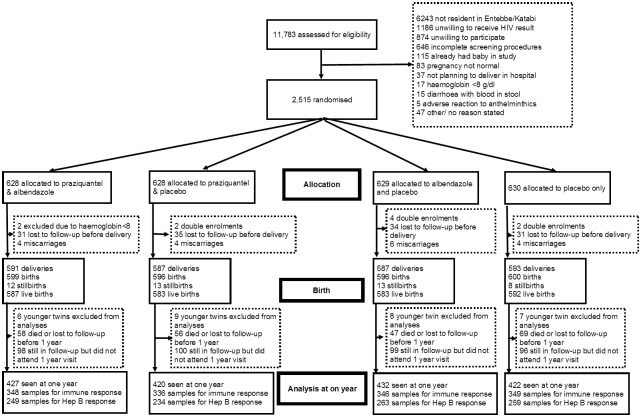
Flow of patients through the trial.

A total of 2507 mothers were enrolled into the trial [7]. We had complete vaccine response data (excluding hepatitis B) for 1379 (55%) mothers and first-born babies: 348 were randomised to albendazole + praziquantel, 346 to albendazole + placebo, 336 to praziquantel + placebo, and 349 to placebos only. Due to limited serum and plasma from infants, samples were unavailable for Hepatitis B assay for 374 of these infants (Fig 1). The demographic characteristics of these 1379 mothers were similar to those who were missing from our analysis. The biggest difference was in maternal malaria infection: this was 9.4% in those included in the analysis and 12.9% in those excluded. Other characteristics were broadly similar: average age was 23.9 years (included) and 23.4 years (excluded); education was “none” or “primary” in 54.7% in the included group and 54.5% in the excluded group. Hookworm infection was 43.2% and 45.3%, and *S*. *mansoni* infection was 17.3% and 19.7% in the included and excluded groups respectively. The sex of the babies was also similar between the included (49.6% female) and excluded groups (47.3% female), as was the parity of the mothers (mean 2.8 in both groups).

Baseline characteristics were broadly balanced between the randomised arms (Table 1). The most common maternal infection among the mothers with vaccine response data was hookworm (43.1%), followed by *Mansonella* (20.9%), *S*. *mansoni* (17.3%), *Strongyloides* (11.7%), malaria (9.4%), *Trichuris* (8.1%) and *Ascaris* (2.1%). We had stool samples for 1235 (89.6%) of one-year olds. The most common parasites detected were *Ascaris* (n = 16) and *Trichuris* (n = 12). We found very low levels of hookworm (n = 4) and *S*. *mansoni* (n = 1).

**Table 1:** Baseline characteristics of mothers whose children were enrolled into the study and provided samples at nine years of age.

Characteristic	Albendazole + praziquantel (n = 348)	Praziquantel only (n = 336)	Albendazole only (n = 346)	Placebo (n = 349)
**Mother's age group**				
**14–19**	68 (20%)	82 (24%)	88 (25%)	72 (21%)
**20–24**	123 (35%)	129 (38%)	125 (36%)	144 (41%)
**25–29**	98 (28%)	78 (23%)	81 (23%)	74 (21%)
**30–34**	41 (12%)	32 (10%)	28 (8%)	44 (13%)
**35+**	18 (5%)	15 (4%)	24 (7%)	15 (4%)
**Mother's education (2 missing values)**				
**None**	11 (3%)	10 (3%)	10 (3%)	16 (5%)
**Primary**	181 (52%)	171 (51%)	182 (53%)	172 (49%)
**Senior**	124 (36%)	117 (35%)	134 (39%)	124 (36%)
**Tertiary**	32 (9%)	37 (11%)	20 (6%)	36 (10%)
**Mother's tribe (2 missing values)**				
**Baganda**	181 (52%)	173 (51%)	176 (51%)	167 (48%)
**Banyankole**	41 (12%)	20 (6%)	34 (10%)	40 (11%)
**Batoro**	11 (3%)	19 (6%)	11 (3%)	21 (6%)
**Basoga**	15 (4%)	8 (2%)	9 (3%)	10 (3%)
**Luo**	15 (4%)	25 (7%)	21 (6%)	18 (5%)
**Banyarwanda**	21 (6%)	15 (4%)	20 (6%)	19 (5%)
**Others**	64 (18%)	76 (23%)	75 (22%)	74 (21%)
**Mother's SES group (22 missing values)**				
**1 Lowest**	16 (5%)	19 (6%)	14 (4%)	19 (6%)
**2**	26 (8%)	25 (8%)	34 (10%)	30 (9%)
**3**	105 (31%)	105 (32%)	94 (28%)	116 (34%)
**4**	101 (29%)	92 (28%)	90 (26%)	102 (30%)
**5**	73 (21%)	68 (21%)	87 (26%)	61 (18%)
**6 Highest**	22 (6%)	21 (6%)	21 (6%)	16 (5%)
**Mother's parity (22 missing values)**				
**1**	87 (25%)	90 (27%)	91 (26%)	86 (25%)
**2–4**	193 (55%)	191 (57%)	199 (58%)	209 (60%)
**5+**	68 (20%)	55 (16%)	56 (16%)	54 (15%)
**Hookworm (22 missing values)**				
**No**	189 (54%)	185 (55%)	214 (62%)	196 (56%)
**Yes**	159 (46%)	151 (45%)	132 (38%)	153 (44%)
**S. mansoni (22 missing values)**				
**No**	292 (84%)	281 (84%)	284 (82%)	284 (81%)
**Yes**	56 (16%)	55 (16%)	62 (18%)	65 (19%)
**Malaria (22 missing values)**				
**No**	321 (92%)	305 (91%)	311 (90%)	313 (90%)
**Yes**	27 (8%)	31 (9%)	35 (10%)	36 (10%)
**Filariasis (22 missing values)**				
**No**	274 (79%)	266 (79%)	279 (81%)	272 (78%)
**Yes**	74 (21%)	70 (21%)	67 (19%)	77 (22%)
**Ascaris (22 missing values)**				
**No**	343 (99%)	328 (98%)	339 (98%)	340 (97%)
**Yes**	5 (1%)	8 (2%)	7 (2%)	9 (3%)
**Trichuris (22 missing values)**				
**No**	320 (92%)	306 (91%)	320 (92%)	321 (92%)
**Yes**	28 (8%)	30 (9%)	26 (8%)	28 (8%)
**Strongyloidiasis (22 missing values)**				
**No**	301 (86%)	297 (88%)	308 (89%)	311 (89%)
**Yes**	47 (14%)	39 (12%)	38 (11%)	38 (11%)

We found no evidence of an effect of randomised treatment on any of the infant vaccine responses (Table 2), nor any evidence for treatment interaction (p>0.1 for all outcomes, S1 Table). In pre-planned sub-group analysis, the only evidence of a differential treatment effect was on DT response in children of mothers who received albendazole: in mothers with hookworm the adjusted geometric mean ratio (aGMR) for albendazole was 0.89 (95 CI% 0.74–1.08) and in mothers without hookworm it was 1.24 (95% CI 1.04–1.47). The p-value for interaction was p = 0.01 (Table 3).

**Table 2:** The effect of randomised maternal treatment on antibody concentrations among infants at one year of age.

Antibody concentrations	Geometric mean		Geometric mean ratio (95% CI)*	Geometric mean		Geometric mean ratio (95% CI)*
	Albendazole	Placebo		Praziquantel	Placebo	
**HiB (g/ml)**	0.89	0.78	1.14 (0.93–1.39)	0.8	0.87	0.92 (0.75–1.13)
**Diphtheria (Dtox IU/ml)**	0.03	0.03	1.07 (0.94–1.22)	0.03	0.03	0.93 (0.82–1.05)
**Hepatitis B (mIU/ml)**	99.94	97.27	1.03 (0.86–1.23)	90.41	106.88	0.85 (0.71–1.01)
**Pertussis toxin (Ptx EU/ml)**	10.78	9.02	1.20 (0.99–1.44)	9.92	9.81	1.01 (0.84–1.21)
**FHA (EU/ml)**	3.1	2.86	1.08 (0.97–1.21)	2.99	2.97	1.01 (0.90–1.13)
**Pertactin (Pm EU/ml)**	11.36	10.97	1.04 (0.91–1.17)	10.96	11.36	0.96 (0.84–1.11)

* Bias-corrected accelerated CIs computed by bootstrapping

**Table 3:** Subgroup analysis of the effect of randomised maternal treatment on antibody concentrations among infants at one year of age in groups according to maternal infection status.

	Albendazole			Praziquantel		
Antibody concentration	Effect of treatment in women with hookworm (aGMR, 95% CI)*	Effect of treatment in women without hookworm (aGMR, 95% CI)*	P-value for interaction	Effect of treatment in women with schistosomiasis (aGMR, 95% CI)*	Effect of treatment in women without schistosomiasis (aGMR, 95% CI)*	P-value for interaction
**HiB (g/ml)**	1.03 (0.77–1.38)	1.22 (0.93–1.61)	0.39	0.98 (0.58–1.63)	0.91 (0.73–1.14)	0.80
**Diphtheria (Dtox IU/ml)**	0.89 (0.74–1.08)	1.24 (1.04–1.47)	0.01	0.88 (0.66–1.18)	0.94 (0.82–1.08)	0.70
**Hepatitis B (mIU/ml)**	0.93 (0.72–1.22)	1.11 (0.88–1.40)	0.33	1.17 (0.76–1.80)	0.79 (0.66–0.95)	0.09
**Pertussis toxin (Ptx EU/ml)**	1.10 (0.86–1.42)	1.27 (1.00–1.61)	0.39	1.03 (0.71–1.51)	1.00 (0.82–1.23)	0.89
**FHA (EU/ml)**	1.17 (0.98–1.41)	1.02 (0.87–1.20)	0.29	1.11 (0.87–1.42)	0.99 (0.86–1.12)	0.44
**Pertactin (Pm EU/ml)**	0.99 (0.83–1.18)	1.07 (0.91–1.26)	0.47	1.03 (0.78–1.38)	0.95 (0.83–1.10)	0.58

* Bias-corrected accelerated CIs computed by bootstrapping. All models are adjusted for randomised treatments, maternal age, gravidity, maternal education, household SES, maternal income, infant sex and previous infant malaria.

For the observational analysis, a total of 1286 mothers-baby pairs had known status for all seven infections of interest and five of the six vaccines (Hepatitis B, n = 940). We found no evidence of different vaccine response results in the excluded records. Similar to the trial analysis, the most common maternal infection in this group was hookworm (43.1%), followed by *Mansonella* (21.2%), *S*. *mansoni* (16.7%), *Strongyloidiasis* (11.6%), malaria (9.2%), *Trichuris* (8.5%) and *Ascaris* (1.9%).

We found no evidence that maternal infections were associated with infant vaccine response except for maternal strongyloidiasis. The aGMR of HiB response for children of mothers with strongyloidiasis was 1.51 times greater (95% CI 1.11–2.01) than children of mothers who were uninfected. For Hep B the increase was by a factor of 1.47 (95% CI 1.11–1.94) and in pertussis response the aGMR was 1.41 (95% CI 1.06–1.88). We found no evidence of an association between any of the maternal exposure groups (number of infections, number of infections alongside malaria, number of infections among mothers without malaria) and any infant vaccine response at one year. Full results are in Table 4.

**Table 4:** Effect of maternal infections on antibody concentrations among infants at one year of age.

	Hib*		Diphtheria*		Hep B*		Pertussis toxin*		FHA*		Pertactin*	
	aGMR	95% CI	aGMR	95% CI	aGMR	95% CI	aGMR	95% CI	aGMR	95% CI	aGMR	95% CI
**Individual infections model**												
**Malaria**	0.833	0.585–1.187	0.926	0.745–1.151	1.296	0.938–1.791	1.224	0.889–1.685	1.039	0.822–1.313	0.886	0.703–1.116
**S. mansoni**	0.952	0.702–1.292	1.107	0.927–1.321	0.993	0.765–1.290	0.912	0.723–1.149	0.96	0.811–1.137	1.032	0.892–1.195
**Hookworm**	0.974	0.789–1.204	1.044	0.904–1.206	1.028	0.847–1.247	0.934	0.758–1.151	1.087	0.951–1.243	1.036	0.911–1.178
**Filariasis**	1.086	0.825–1.428	1.009	0.864–1.178	1.084	0.866–1.358	0.956	0.771–1.185	0.961	0.826–1.117	0.975	0.832–1.143
**Ascaris**	1.315	0.562–3.079	1.283	0.832–1.980	1.109	0.552–2.227	1.468	0.732–2.944	1.295	0.804–2.086	0.975	0.631–1.509
**Trichuris**	0.684	0.475–0.986	0.901	0.713–1.138	0.869	0.631–1.195	1.042	0.746–1.455	1.029	0.800–1.323	0.967	0.788–1.187
**Strongyloidiasis**	1.514	1.110–2.066	1.151	0.919–1.441	1.469	1.112–1.940	1.41	1.055–1.884	0.977	0.790–1.207	1.138	0.951–1.361
**Number of maternal infections (including malaria) model**		P = 0.565		P = 0.112		P = 0.251		P = 0.236		P = 0.192		P = 0.840
**None**	-		-		-		-		-		-	
**1**	0.875	0.687–1.114	0.908	0.770–1.071	1.006	0.790–1.281	0.947	0.773–1.160	0.913	0.791–1.055	1.044	0.897–1.214
**2**	1.036	0.760–1.412	0.957	0.793–1.155	1.225	0.927–1.620	0.906	0.701–1.169	1.053	0.891–1.243	0.996	0.834–1.189
**3 or more**	0.99	0.659–1.485	1.187	0.936–1.507	1.28	0.881–1.861	1.26	0.895–1.773	1.106	0.876–1.397	1.09	0.858–1.386
**Number of helminths model**		P = 0.434		P = 0.638		P = 0.112		P = 0.600		P = 0.278		P = 0.314
**None**	-		-		-		-		-		-	
**Malaria alone**	0.653	0.378–1.129	0.862	0.569–1.307	1.15	0.564–2.346	1.109	0.578–2.128	1.061	0.622–1.812	1.151	0.750–1.767
**+ 1 helminth**	0.832	0.451–1.535	0.83	0.595–1.159	1.766	1.089–2.864	1.096	0.647–1.854	1.279	0.850–1.923	0.771	0.557–1.067
**+ 2 or more**	0.834	0.436–1.594	1.02	0.688–1.512	1.338	0.746–2.399	1.402	0.854–2.302	0.803	0.593–1.088	1.104	0.729–1.674
**Number of helminths (mothers without malaria) model**		P = 0.626		P = 0.045		P = 0.411		P = 0.418		P = 0.111		P = 0.880
**None**	-		-		-		-		-		-	
**1 helminth**	0.887	0.678–1.161	0.914	0.773–1.079	1.003	0.786–1.279	0.933	0.747–1.165	0.901	0.771–1.054	1.034	0.884–1.209
**2 helminths**	1.063	0.759–1.488	0.979	0.810–1.185	1.158	0.894–1.500	0.877	0.676–1.139	1.002	0.848–1.184	1.043	0.874–1.246
**3 or more**	1.049	0.671–1.641	1.291	0.997–1.671	1.308	0.858–1.993	1.218	0.840–1.766	1.291	0.945–1.764	1.121	0.837–1.502

* All models were additionally adjusted for randomised treatments, maternal age, gravidity, maternal education, household SES, maternal income, infant sex and previous infant malaria. All reported CIs are computed using bootstrap with a bias-corrected accelerator.

## Discussion

We found no evidence of enhanced vaccine responses among infants of infected mothers who were treated for helminths during pregnancy, nor evidence of a suppressive effect of prenatal exposure to maternal parasitic infections on infant vaccine responses, in this cohort of mothers and infants in Uganda. Such possible effects as were observed were indicative of *enhanced* responses for a number of vaccines in the infants of mothers identified as having strongyloidiasis, and of *reduced* DT responses in the infants of mothers with detectable hookworm infection who were treated with albendazole.

The primary strength of our study is the randomised, controlled intervention during pregnancy. Hookworm infection was treated effectively by albendazole (declining from over 40% prevalence before treatment to 5% after delivery among albendazole treated women) and schistosomiasis was treated effectively by praziquantel (declining from about 18% to 5%) while *Mansonella* and *Strongyloides* were unaffected by the treatment [15]. From a simplistic perspective, the lack of effect of maternal treatment on vaccine responses among infants of women infected with hookworm and *S*. *mansoni* implies either that prenatal exposure to these helminth species has no important effect on the infant response to unrelated vaccines (and hence perhaps to unrelated infections), or that the impact of prenatal exposure is established prior to the second trimester and cannot be reversed thereafter. Of note, Malhotra and colleagues treated all mothers with albendazole (for nematodes) during pregnancy; we, and Malhotra and colleagues, treated all mothers with sulfadoxine / pyrimethamine (for malaria); the effects described in each study were necessarily those that occur despite, or in the context of, these interventions [5].

However, the truth of the matter seems to be that the impact of prenatal parasitic infections on infant vaccine responses is complex and depends at least on characteristics of both the parasitic infection and the vaccine, and on the nature of the desired, protective vaccine response. A study from Ecuador, in accord with our results, showed no association between maternal geohelminths and infant IgG responses to DT, TT, PT, measles, rubella or HiB [16], but several studies have now indicated a net enhancement of infant vaccine responses following exposure to certain maternal infections, including *Trypanosoma cruzi* for BCG, DT, TT and Hep B [17], maternal intestinal helminth infections and the IgA response to rotavirus and polio (in the Ecuador study [16]), as well as our result for strongyloidiasis and PT, HiB and Hep B. Meanwhile, Malhotra and colleagues have shown that, for malaria and lymphatic filariasis (and, in an earlier study, schistosomiasis [18]), the impact of maternal infection on infant vaccine response depends upon whether or not the infant was sensitised to the parasitic infection in utero: compared to unexposed infants, malaria sensitised infants showed an increase, and malaria tolerised infants a decrease, in the response to DT [5]–this may contribute to a neutral net effect in studies which do not make the same distinction. Like us, Malhotra and colleagues observed no effect of pre-natal exposure to parasitic infections on infant responses to most of the EPI vaccines. The principal exception was HiB and, interestingly, although individual maternal infections were associated with reduced responses, additional infections tended to reverse this effect. Our findings for HiB followed a similar pattern, although the associations were not statistically significant.

Our observation of an enhanced response to DT amongst infants of mothers without hookworm who received albendazole was surprising, and may be a chance finding given that subgroup analyses were conducted and multiple comparisons were made, with no formal adjustment in statistical interpretation. However, this result accords with our previous findings of an enhanced IFN-γ response to BCG, an enhanced IL-13 response to TT, and an enhanced risk of infantile eczema in the same group [7, 19] and suggests a pro-inflammatory effect of albendazole, in the absence of maternal hookworm, which may be a direct effect of the drug, or mediated by effects on other co-infections. We think it unlikely that these results represent an effect of albendazole on light, undetected hookworm infections: as we have previously reported, a proportion of mothers in the albendazole placebo group had three samples examined before treatment was given post-delivery, evaluation of which increased the prevalence of hookworm in this group by only 6% (from 45% to 51%) [19].

A net adverse effect of prenatal exposure to maternal parasitic infections on the induction of immune responses by vaccines given to the offspring would imply a net adverse effect on the infant’s ability to respond to pathogens, also. This would be expected to result in increased neonatal or infant mortality. However, an initial result suggesting that anthelminthic treatment during pregnancy had benefits for infant mortality [20] has not been substantiated in controlled trials [7, 21]. By contrast, there is considerable evidence that intervention against malaria during pregnancy has benefits for infant mortality [22]. While this may be mediated largely by prevention of the major effects of malaria on placental function and fetal growth, and by effects on infant susceptibility to malaria itself [23, 24], an impact on vaccine responses and on susceptibility to heterologous infections may contribute. This accords with the relatively prominent suppressive effect of maternal malaria described in Malhotra’s study, with a reported suppressive effect of prenatal exposure to malaria on the infant response to BCG, described in The Gambia [25] and with our own previous finding of an association between maternal malaria and reduced infant antibody response to measles immunisation [26].

A limitation of our study was that we included just 55% of eligible infants due to incomplete data. However, the mother-baby pairs that were excluded were similar in known characteristics to those included in the analysis. Further infants were missing from the Hep B analysis. A limitation of the observational component of our study was the classification of maternal infection status based on a single blood or stool sample. This would substantially underestimate and misclassify malaria exposure, which is best assessed by placental histology, and more sensitively assessed by polymerase chain reaction (PCR) assays. The use of a single stool result would also result in misclassification for hookworm or *S*. *mansoni* exposure [27, 28] and hence may have obscured relevant associations. We have previously reported that in this study the sensitivity of one stool sample compared with three stool samples was 89% for hookworm infection and 66% for schistosomiasis [7]. This limitation does not apply to evaluation of *Mansonella* exposure which showed 96% agreement between samples taken in pregnancy and after delivery in this cohort (2077 of 2162 mothers for whom samples were available at both time points). Our classification of exposure differed markedly from the classification used by Malhotra and colleagues who included microscopy and PCR on placental and cord blood for malaria, and assays of circulating antigen and IgG4 for *Schistosoma haematobium* and *Wuchereria bancrofti*. The use of IgG4 detection as a marker of active infection is of possible concern as levels may be higher among individuals with a regulatory bias in their immune response [29]. Contrasting with the time-course described by Malhotra and colleagues, we had data on vaccine-specific antibody responses only at age one year, but this was a time point at which many of the effects observed by Malhotra and colleagues were evident, so comparable results might have been expected. Although other markers or measured timepoints may be more desirable in terms of assessing true protection against disease (such as neutralisation or functional opsonophagocytic assays [14]), many of these other assays are more prone to inter-observer error. Our analysis of the unstimulated antibody concentrations maintains consistency between studies and provides observations relating to immunological responses rather than chances of protection. Furthermore, we had data on a different set of helminth infections to those used by Malhotra. It could be that particular helminths are more important in determining vaccine responses of infants, or that there are interaction effects which we have not explored. This paper contains a large number of estimates, confidence intervals and hypothesis tests. Due to these multiple comparisons, it is possible that some associations which appear statistically significant are in fact due to chance alone. These results should therefore not be considered definitive, but should instead be seen as evidence to be considered alongside other studies in this field.

From a public health perspective, the additional results that we contribute here accord with our previous findings [7, 26] and suggest that, whatever its benefits, routine anthelminthic treatment during pregnancy is not likely to result in improved infant vaccine responses.

## Supporting information

S1 Table Unadjusted analysis of treatment effect allowing an interaction between randomised treatments. (DOCX)
