# Supplementary material for: The impact of prenatal exposure to parasitic infections and to anthelminthic treatment on antibody responses to routine immunisations given in infancy: Secondary analysis of a randomised controlled trial
Source: PLoS Negl Trop Dis. 2017 Feb 8;11(2):e0005213. doi: 10.1371/journal.pntd.0005213 (PMC5298230; doi:10.1371/journal.pntd.0005213)
Supplement: S1 Table — (DOCX) [file pntd.0005213.s001.docx]

| **Antibody concentrations** | **Geometric mean ratio (95% confidence interval)** | | | | **P-value for interaction** |
| --- | --- | --- | --- | --- | --- |
|  | **Albendazole among praziquantel placebo group** | **Albendazole among active praziquantel group** | **Praziquantel among albendazole placebo group** | **Praziquantel among active albendazole group** |  |
| **HiB (g/ml)** | 1.27 (0.96-1.68) | 1.02 (0.76-1.35) | 1.03 (0.78-1.37) | 0.82 (0.62-1.09) | 0.27 |
| **Diphtheria (Dtox IU/ml)** | 1.15 (0.96-1.37) | 1.00 (0.84-1.20) | 1.00 (0.83-1.19) | 0.87 (0.73-1.04) | 0.29 |
| **Hepatitis B (mIU/ml)** | 0.91 (0.71-1.16) | 1.18 (0.91-1.53) | 0.74 (0.57-0.95) | 0.96 (0.75-1.23) | 0.15 |
| **Pertussis toxin (Ptx EU/ml)** | 1.30 (1.02-1.65) | 1.10 (0.86-1.40) | 1.10 (0.86-1.40) | 0.93 (0.73-1.18) | 0.33 |
| **FHA (EU/ml)** | 1.16 (0.98-1.36) | 1.01 (0.86-1.19) | 1.08 (0.91-1.27) | 0.94 (0.80-1.11) | 0.25 |
| **Pertactin (Pm EU/ml)** | 1.11 (0.94-1.32) | 0.96 (0.81-1.14) | 1.04 (0.87-1.23) | 0.90 (0.76-1.06) | 0.25 |

**Supplementary Table 1. Unadjusted analysis of treatment effect allowing an interaction between randomised treatments**
